# Blepharospasm: Update on Epidemiology, Clinical Aspects, and Pathophysiology

**DOI:** 10.3389/fneur.2016.00045

**Published:** 2016-03-31

**Authors:** Josep Valls-Sole, Giovanni Defazio

**Affiliations:** ^1^EMG and Motor Control Section, Neurology Department, Hospital Clinic, University of Barcelona, Barcelona, Spain; ^2^Department of Basic Medical Sciences, Neurosciences and Sensory Organs, “Aldo Moro” University of Bari, Bari, Italy

**Keywords:** blepharospasm, blink reflex, excitability testing, neuroimaging, epidemiology

## Abstract

Blepharospasm (BSP) is a rather distressing form of focal dystonia. Although many aspects of its pathophysiological mechanisms are already known, we lack fundamental evidence on etiology, prevention, and treatment. To advance in our knowledge, we need to review what is already known in various aspects of the disorder and use these bases to find future lines of interest. Some of the signs observed in BSP are cause, while others are consequence of the disorder. Non-motor symptoms and signs may be a cue for understanding better the disease. Various cerebral sites have been shown to be functionally abnormal in BSP, including the basal ganglia, the cortex, and the cerebellum. However, we still do not know if the dysfunction or structural change affecting these brain regions is cause or consequence of BSP. Further advances in neurophysiology and neuroimaging may eventually clarify the pathophysiological mechanisms implicated. In this manuscript, we aim to update what is known regarding epidemiology, clinical aspects, and pathophysiology of the disorder and speculate on the directions of research worth pursuing in the near future.

## Introduction

Blepharospasm (BSP) is a form of focal dystonia that manifests with spasms of the eyelids, involuntary closure of the eye, and enhanced spontaneous blinking, or any combination of the previous ones. We have advanced in our knowledge of the disorders since the first descriptions of BSP as a form of dystonia (1, 2). However, there are still many unknown aspects in the generation of the disorder and unclear points in the pathophysiological mechanisms of the various forms of focal dystonia. BSP is particularly distressing. Patients may be functionally blind and unable to pursue a normal social life, with plenty of emotional and behavioral consequences. In this manuscript, we aim to update what is known regarding epidemiology, clinical aspects, and pathophysiology of the disorder and speculate on the directions of research worth pursuing in the near future.

## Review of Known BSP Phenomenology

### Epidemiology, Clinical Aspects, and Diagnostic Tools

Although BSP is now recognized as one of the most common forms of adult-onset dystonia, it is thought to be rare, affecting about 16–133 cases per million (3). By most studies, BSP seems to be less prevalent than primary cervical dystonia (CD) but in Japan, and in Italy too, the trend is reversed, BSP being more frequent than CD (3). Peculiar characteristics of the BSP are female preference (3), peak age at onset between the fifth and the seventh decade, and a greater tendency to spread to adjacent body parts (usually within the first 5 years of history) than cervical and upper limb dystonia (4). BSP patients may also have tremor in the head or upper limbs (5).

#### Motor Phenomenology

Blepharospasm is characterized by stereotyped, bilateral, and synchronous spasms of the orbicularis oculi muscles. Spasms may be brief or sustained and may induce narrowing or closure of the eyelids. Other relevant manifestations include: sensory trick that can transiently improve eyelid spasms in about half of the patients (6), associated apraxia of eyelid opening (7), and increased spontaneous blink rate (8). Whether increased blinking may preceed BSP is an open question for future studies (9).

A diagnostic algorithm has been developed, based on motor manifestations of stereotyped, bilateral, and synchronous orbicularis oculi spasms, identification of sensory trick or, alternatively, increased blinking (5, 10, 11), which yielded 93% sensitivity and 90% specificity in distinguishing BSP from other conditions of involuntary lid closure such as eyelid tics, hemifacial spasms, facial chorea, apraxia of eyelid opening, frequent blinking, and lid ptosis due to myasthenia or other causes. The new severity scale was based on six clinical aspects, including degree and duration of eyelid closure, frequency of spasms, presence of apraxia of eyelid opening, occurrence of spasms during writing and increased blinking. The scale is suitable to assess BSP severity in clinical practice and research, yielding moderate to almost perfect reliability and acceptable clinimetric properties (5, 11).

#### Non-Motor Phenomenology

Several non-motor manifestations may be more frequent in BSP patients than in healthy or disease controls. They belong to four domains: sensory symptoms, psychiatric disorders, sleep disorders, and cognitive disturbances. Symptoms belonging to the sensory domain include burning sensation and grittiness in the eye, dry eye, and photophobia. Theoretically, eye symptoms may be part of the spectrum of BSP but they can also result from eye diseases of the anterior segment of the eyes, which have been identified with 77% sensitivity and 94% specificity in BSP patients (12).

Psychiatric alterations include depression, anxiety, and obsessive–compulsive disorders (13, 14). The finding is not specific because psychiatric disturbances, mostly depression, are also more frequent in patients with various forms of focal dystonia than in healthy controls. Theoretically, psychiatric disorders may be part of the clinical spectrum of the disease or secondary to the dystonia-induced disability. By most studies, depression appears to represent a feature of primary dystonia, whereas other psychiatric abnormalities have a less certain relationship and need additional evaluation.

Sleep impairment may be another feature of BSP (15). It seems to be independent of motor severity but rather correlated with depression. Therefore, it is not clear at present if there is a primary sleep abnormality in dystonia. Further studies are warranted.

There is little evidence of alteration of cognitive functions in idiopathic dystonia. Nevertheless, a recent study showed that non-depressed and non-demented patients with cranio-CD and normal IQ may have impairments in several specific cognitive domains including working memory, information processing speed, and set-shifting capacity (16). Altered cognitive measures were independent of the clinical expression of dystonia.

### Neuroimaging

#### Structural

As in other forms of focal dystonia, BSP is considered to originate from a dysfunction in basal ganglia circuitry, although other brain and brainstem circuits can also be involved. Lesions causing BSP have been identified in various sites. In the analysis of their own cases, Khooshnoodi et al. (17) found 18 out of 1114 patients with BSP to have brain lesions that could account for their symptoms (1.6%). In a total of 48 cases (30 of them extracted from the literature), these authors reported lesions in several parts of the brain, including the thalamus in 12, lower brainstem in 11, basal ganglia in 9, cerebellum in 9, midbrain in 7, and cortex in 1. These observations have contributed to the idea of a widely distributed network of regions where lesions can potentially lead to BSP.

Although evidence for lesions in patients with BSP is scarce, researchers on brain neuroimaging have found group abnormalities in various sites also. The first to report on volumetric abnormalities in idiopathic focal dystonia was Black et al. (18). These authors studied 13 patients (5 with BSP and 8 with hand dystonia) and found that the gray-matter volume (GMV) of the putamen was about 10% larger in patients than in healthy controls. The authors speculated on the possibility that putaminal GMV change could be a response to dystonia if not related to its cause. A putaminal increase in GMV was confirmed in 16 patients with BSP by Etgen et al. (19) who used voxel-based morphometry rather than a direct analysis of a region of interest. These authors observed also decreased GMV in the left inferior parietal lobe, which correlated significantly with duration of botulinum toxin treatment and suggested a crucial role for the putamen in the pathophysiology of focal dystonia and secondary changes related with the tonic spasms and their botulinum toxin treatment for the left parietal region.

However, not all studies confirmed the increase in volume and hyperactivity in the striatal region. Obermann et al. (20) found a decrease rather than an increase in GMV in the putamen and thalamus bilaterally, whereas they reported increased GMV in the caudate head and the cerebellum, also bilaterally. In this study, though the authors examined patients with BSP (11) together with patients with CD (9) in an attempt to find common sites of involvement shared by these two forms of focal dystonia. In any case, they concluded that the morphometric changes found were located within structures important for sensorimotor integration and motor control, pointing out to a functional damage that, with time, may lead to structural changes. Other authors reported no significant change in microstructure of basal ganglia using diffusion tensor imaging (7) or voxel-based morphometry (21). The latter study is relevant for reporting, apart from the absence of changes in the basal ganglia, greater GMV in BSP patients than in healthy volunteers in the right middle frontal gyrus and the reverse pattern (i.e., smaller GMV in patients than in healthy volunteers) in the left postcentral gyrus and left superior temporal gyrus. Therefore, these authors concluded that patients may have cortical but no basal ganglia changes. It is difficult to know the cause of the differences in the findings reported by Martino et al. (21) and those reported by Etgen et al. (19). Martino et al. (21) argued that the number of patients they studied was larger: 25 vs. 16 studied by Etgen et al. (19). However, it is difficult to believe that nine patients can account for the differences reported. Other factors have to be taken into account to explain the different findings reported so far, such as age at disease onset, disease duration, presence of dystonia spread to other body sites, dystonia severity, and duration and mean dose of botulinum toxin treatment. In none of these variables, the authors found a correlation with the neuroimaging findings but there can still be some influence from these variables enough to tilt the subtle abnormalities of BSP patients toward one or another direction.

Involvement of the cortex and the corticonuclear tract was supported by another morphometric study (22) that showed a significant decrease in GMV in the facial portion of the left primary motor cortex and right anterior cingulate of BSP patients when compared to healthy volunteers. An interesting comparison was made by Ramdhani et al. (23) between patients with task-specific (laryngeal dystonia and writer’s cramp) and non-task specific (BSP and CD) forms of dystonia. They found that GMV changes in task-specific dystonia involved the brain regions responsible for sensorimotor control during writing and speaking, such as primary somatosensory cortex, middle frontal gyrus, superior/inferior temporal gyrus, middle/posterior cingulate cortex, and occipital cortex as well as the striatum and cerebellum (lobules VI–VIIa), whereas those in non-task-specific dystonia were limited to the left cerebellum (lobule VIIa) only. The authors concluded that these two forms of dystonia may have different pathophysiological mechanisms, may be precipitated by different triggers and express in neuroimaging as distinct microstructural patterns. The findings of Ramdhani et al. (23) are indeed stimulating for going further on structural and functional analysis of the brain in different forms of dystonia. They also put on show the contribution of the cerebellum as a key structure in the brain dystonia network (22, 24).

Indeed, the latest findings in neuroimaging studies have revealed microstructural abnormalities in the cerebellum. Yang et al. (25) studied diffusion tensor imaging and voxel-based fractal anisotropy in 9 patients with BSP alone, compared with 11 patients with BSP plus oromandibular dystonia. BSP patients showed significant FA reductions in the left anterior lobe of cerebellum that correlated negatively with disease severity, while patients with BSP and oromandibular dystonia showed an increase of FA in the right parietal lobe that correlated negatively with disease duration. Other abnormalities were also found in areas around the right precuneus, lentiform nucleus, and insula in different combinations in the two groups of patients. The authors concluded that white-matter changes outside the basal ganglia may present trait features that are specific for individual phenotypes of dystonia.

#### Functional

Many functional abnormalities have been reported in BSP patients. In 1995, Smith et al. (26) used [18F]fluorodeoxyglucose positron emission tomography (18-FDG PET) to find abnormally reduced medial frontal lobe glucose metabolism in four patients with apraxia of lid opening. A similar study on 10 patients with BSP showed increased glucose metabolism in the striatum and thalamus (27). Two interesting additional observations were made in that study: five patients were investigated before and after treatment of muscle spasms with botulinum toxin and showed similar results, indicating that the abnormalities were intrinsic of the disorder and not the consequence of increased muscle activity. On the other side, the authors did not find any significant correlation between severity of the spasms and the degree of striatum or thalamus hypermetabolism. Abnormalities in basal ganglia and frontal cortex have been reported since then, using various functional neuroimaging techniques, in various studies of patients with BSP (28). Kerrison et al. (29) reported cortical areas of increased glucose metabolism (inferior frontal gyri, right posterior cingulate gyrus, left middle occipital gyrus, fusiform gyrus of the right temporal lobe, and left anterior cingulate gyrus) and others with decreased glucose uptake (a region ventral to the area of increased glucose metabolism in the frontal inferior gyri). They also found increased glucose uptake in the right caudate and decreased glucose uptake in the left inferior cerebellar hemisphere and thalamus. Hutchinson et al. (30) examined PET in six BSP patients during sleep to avoid the possible confounding effect of spasm-related muscular contractions. They found that during sleep, patients showed frontal hypometabolism in a region associated with cortical control of eyelid movements while they showed hypermetabolism of the cerebellum and pons during wakefulness, when they exhibited involuntary muscle contractions. Apart from that, network analysis demonstrated overactivity of the lentiform nuclei, cerebellum, and the supplementary motor regions, reported by the same authors previously to be a pattern of abnormalities associated with other forms of dystonia. In a similar line of reasoning, Suzuki et al. (31) used also 18-FDG PET to investigate cerebral glucose metabolism in BSP patients whose spasms were suppressed by botulinum toxin injections. They found significant increase in glucose metabolism in the thalamus and pons that they interpreted as an expression of a compensatory change, common to pathophysiological mechanisms in other types of focal dystonia. The same authors have recently reported that the increase in putaminal glucose metabolism shown using 18-FDG PET in patients with essential BSP may separate them from those with drug-induced BSP who did not show such increase. A correlation between severity of the spasm and the intensity of increased glucose metabolism in the thalamus was reported by Murai et al. (32) in a study of a single patient who underwent five PET sessions during a follow-up of 22 months. An interesting approach was taken by Emoto et al. (33), who used 18-FDG PET to characterize photophobia in BSP patients. These authors found that patients with photophobia had significant hypermetabolism in the thalamus, while those without photophobia had significant hypometabolism in the superior colliculus. These findings may underlie mechanisms for the increase in the blinking rate in BSP patients with photophobia.

A functional magnetic resonance imaging (fMRI) study with analysis of the blood oxygenation level-dependent signal was done during spasms by Schmidt et al. (34). Healthy volunteers were asked to make a voluntary closing of their eyelids that was to be contrasted with rest, while patients were requested to press a button to mark the onset of their spasms. The authors reported on various areas activated with blinking, common to patients and healthy subjects, such as frontal and parietal operculum, supplementary motor area, primary sensorimotor cortex, various visual areas, and the cerebellum. The site where differences were observed was a subregion of the putamen, where the authors found hyperactivation in patients in comparison to healthy volunteers. Baker et al. (35) reported on the fMRI study of spontaneous and voluntary blinking in five BSP patients and five healthy subjects. The authors found greater activation in BSP patients than in control subjects in the anterior visual cortex, anterior cingulate cortex, primary motor cortex, central region of the thalamus, and superior cerebellum. Therefore, their findings suggested the existence of a hyperactive cortical circuit linking visual cortex, limbic system, supplementary motor cortex, cerebellum, and supranuclear motor pathways innervating the periorbital muscles. The existence of an abnormal default mode network implying the cortico-striato-pallido-thalamic loop has been suggested by Zhou et al. (36) after a fMRI study of resting state and voxel-based analysis of amplitude of low-frequency fluctuations.

Dopamine binding has been also examined in BSP. The first report was made in 1997 by Perlmutter et al. (37) who used PET to measure binding of radioligand [18F]spiperone in putamen in 21 patients and 13 healthy subjects to find decreased dopamine D2-like binding in 29% in dystonic patients. This was confirmed by Horie et al. (38), who found decreased binding in the entire striatal region (by a similar percentage in caudate and anterior and posterior putamen). The authors speculated on the possible mechanism relating dopamine ligand striatal deficit and decreased inhibition in BSP, typical of all forms of dystonia.

### Neurophysiology

Dystonia is a functional disorder, and therefore, neurophysiology is a key methodology for its study, both for recognizing the underlying pathophysiological mechanisms and providing cues for the differential diagnosis when clinical signs and symptoms are not sufficiently clear. In the case of BSP, the most useful neurophysiological test is the blink reflex, which can be used to examine the excitability of brainstem interneurons, modulated in turn by circuits feeding on basal ganglia output signals (39–41).

#### Electromyography

The paradigmatic feature of dystonia is cocontraction between antagonistic muscles in attempts to perform a discrete movement. Therefore, one of the key methods for the documentation of abnormalities requires electromyographic recording of antagonistic muscles. This is not feasible in the muscles controlling the eyelid, the levator palpebrae, being unavailable to non-invasive electromyography recordings. A few authors have recorded from the levator palpebrae with needle electrodes showing the expected reduction of reciprocal inhibition (42, 43).

Simple observation of spontaneous blinking allows determining the rate of spontaneous blinking and the strength and duration of eye closure, measures that are rather useful for the characterization of BSP. Additional information can be obtained by recording the EMG activity from the orbicularis oculi with a pair of electrodes attached over the skin overlying the muscle at the lower eyelid. In healthy subjects, spontaneous blinking results from a brief phasic activation of the orbicularis oculi and relaxation of the levator palpebrae, but in BSP patients, the amount of EMG activity in the orbicularis oculi is usually markedly increased (41, 44, 45).

#### Blink Reflex

The study of reflex blinking is an important aspect of the evaluation of patients with neurological disorders. In the clinical context, gentle tapping of the forehead is the most common method to induce reflex blinking. If done repeatedly, clinicians are able to assess the inhibitory control of the reflex (the Myerson’s maneuver). A startling stimulus, whether auditory, visual, or somatosensory, elicits reflex blinking as a form of eye protection (41, 46). However, the method that has been mostly used for the assessment of reflex blinking is the electrical activation of the supraorbital nerve while recording the EMG responses of the orbicularis oculi (47), which is conventionally known as the blink reflex. The electrically induced trigemino-facial blink reflex consists of two separate components: an early ipsilateral R_1_ and a late bilateral R_2_. R_1_ is a pontine reflex, while R_2_ is relayed through a more complex route including the pons and lateral medulla. The fact that unilateral stimuli give rise to bilateral responses permits separate assessment of the afferent and the efferent arms of the reflex circuit. Additionally, the blink reflex can be used to examine various functions that are either integrated in, or mediated by, the brainstem. If no peripheral nerve lesions interfere with the recording, the study of blink reflex becomes a useful technique for the assessment of supranuclear control of brainstem interneuronal excitability (41).

**Figure 1:**
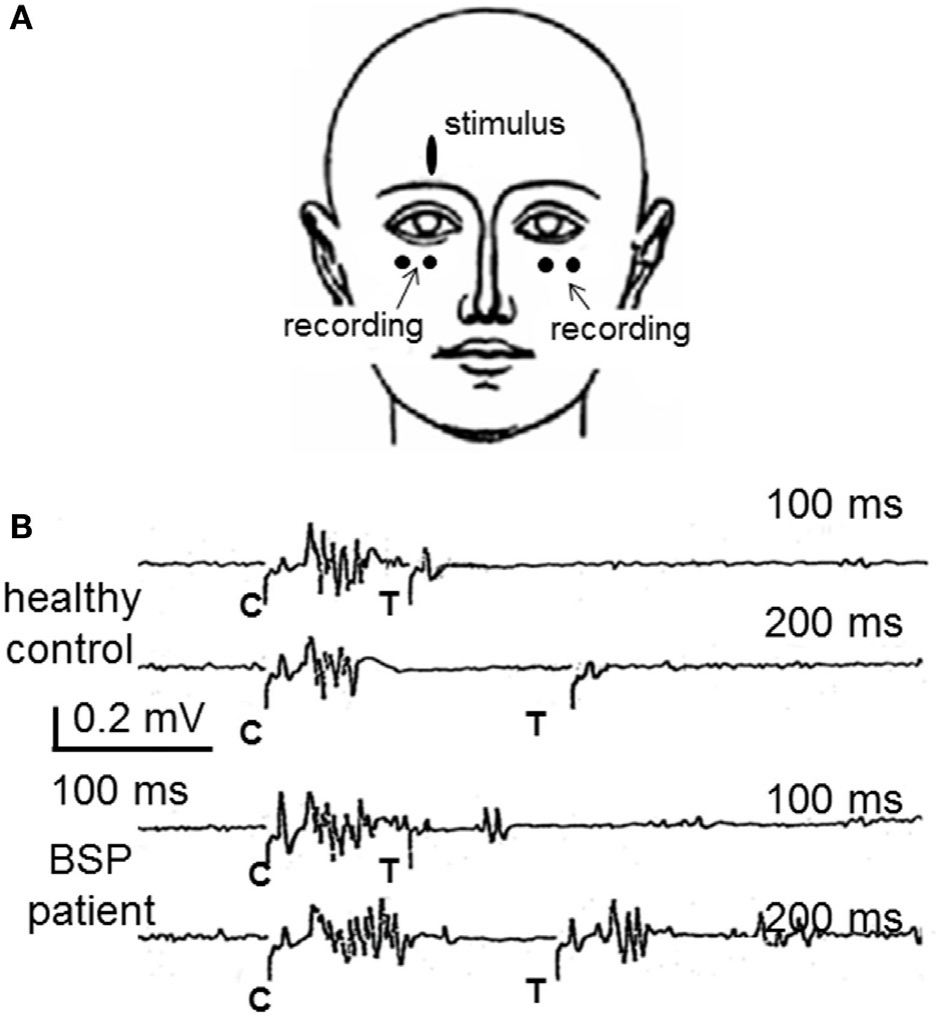
**The study of the blink reflex excitability recovery curve in blepharospasm**. **(A)** The most frequently used method to examine patients with BSP is the recording of blink responses by surface electrodes attached over the orbicularis oculi muscles. **(B)** Partial results from a study of blink reflex excitability recovery in a healthy control and a BSP patient. Pair of supraorbital nerve stimuli are applied either 100 or 200 ms apart. The conditioning stimulus (C) elicits the typical blink reflex, composed by R_1_ and R_2_ responses. The test stimulus (T) elicits no R_2_ response in the healthy control, which reflects the normal lack of excitability at such short intervals, whereas they elicit an R_2_ response in the patient, larger with the interval of 200 ms, reflecting the enhanced blink reflex excitability, characteristic of BSP. Part **(A)** of this figure was previously published as part of Figure 19.6 of Ref. (45) and is reproduced with permission from Elsevier. Part **(B)** of this figure was previously published as Figure 1 in Valls-Sole’s “Assessment of excitability in brainstem circuits mediating the blink reflex and the startle reaction,” published in Ref. (41), and is reproduced with permission from Elsevier.

The best-known method for the assessment of blink reflex excitability is the paired shock technique, which consists in applying pairs of supraorbital nerve stimuli of the same intensity (48). The first stimulus (conditioning) induces a transient change in the excitability of reflex circuits, and the second stimulus (test), delivered at varying inter-stimulus intervals with respect to the first, is used as the probe stimulus. The size of the response elicited by the test stimulus is expressed as a percentage of the response to the conditioning stimulus, and a *X*–*Y* graph of excitability recovery can be represented for all intervals tested (usually between 100 and 1000 ms). The R_2_ response is usually completely abolished from 0 to 200–300 ms, then very slowly recovers, reaching about 30–50% at the 500 ms interval and 70–90% at the 1500 ms interval in healthy volunteers. Blink reflex excitability is assumed to be under the control of rostral structures, including the basal ganglia (39, 40) and is abnormally enhanced in patients with BSP, which shows as a shift to the left in the excitability recovery curve. The Figure 1 shows the methods and results in a single case.

Blink reflex excitability enhancement is not specific for dystonia, and, therefore, the test cannot be used for the diagnosis, although the data obtained may reinforce clinical suspicion. Interestingly, however, it has been reported normal in patients with psychogenic dystonia (49), which makes the test clinically very useful. In fact, blink reflex excitability depends on excitatory inputs reaching brainstem interneurons in the trigemino-facial pathway. One such input is a startling auditory stimulus (SAS). This generalized reflex response, and the more discrete auditory blink reflex, is mediated by the nuclei of the reticular formation. A regulatory step in the pathway is likely the pedunculopontine tegmental nucleus, which also receives inputs from the internal pallidum and other basal ganglia nuclei. The pedunculopontine nucleus has an inhibitory action on the startle blinking, and one way to document the inhibitory control of the blink reflex is prepulse inhibition (50, 51).

Prepulse inhibition is defined as the inhibitory effect of a stimulus (weak enough not to induce a reflex response by its own) on the response to another, suprathreshold, stimulus. This effect is considered to be due to the attentional shift required to process the information brought about by the prepulse. Prepulse inhibition has been found abnormal in patients with BSP, particularly in those who cannot find relief of their symptoms using a “geste antagonistique” (52). The abnormalities in prepulse inhibition show functional impairment of a circuit that is different from that of the blink reflex excitability recovery curve (53).

An animal model of BSP has been reported in which a 6-hidroxi-dopamine lesion of the striatum together with a peripheral nerve lesion may lead to enhanced reflex gain (54, 55). This may apply to humans with some predisposition to develop dystonia, where unilateral peripheral facial nerve lesions lead to enhancement of blink reflex excitability recovery in the side contralateral to paresis. That such changes in the contralateral facial nerve are related to the afferent input from the cornea of the paralyzed side was first suggested by Chuke et al. (56), who found that the BSP-like sustained contraction of the facial muscles of the side contralateral to the paralysis was relieved by helping to close the eye with a weight added to the paralyzed eyelid. Further evidence in the same direction was reported by Manca et al. (57), who analyzed the size differences between the responses recorded in the non-paralyzed side to ipsi- and contralateral supraorbital nerve electrical stimulation. These are examples of maladaptive plastic changes in the control of brainstem excitability that, in predisposed subjects, may lead to clinical expression of dystonia.

#### Other Neurophysiology-Based Recordings

Little information exists on direct neurophysiological recordings from the structures supposedly dysfunctional in BSP, i.e., the basal ganglia, thalamus, brainstem, or cerebellum. Deep brain stimulation (DBS) has been used scarcely to treat BSP patients because significant symptomatic alleviation is usually provided by injections of botulinum toxin in the orbicularis oculi muscles. The few reports published on DBS in BSP patients (actually in patients combining BSP and oromandibular dystonia) deal with clinical and epidemiological aspects (58–60). Special mention should be given to the report of a single patient by Foote et al. (61). In this case, the authors reported an increase in single cells firing rates in the GPi of the right side, which may relate to the hyperactivity reported with neuroimaging studies. An interesting aspect is that the firing rate was less increased in the left side, operated 6 months after the right side, indicating effect of DBS beyond the site of implantation. DBS may not always be beneficial but sometimes may worsen symptoms in BSP patients (62).

The blink reflex can be elicited not only by trigeminal stimuli but also by other somatosensory stimuli applied elsewhere in the body, the so-called somatosensory blink reflex (63). This type of reflex has been also reported to show an abnormal excitability enhancement in patients with BSP (64), which challenges the hypothesis of the influence of the basal ganglia over trigeminal neuronal excitability as the cause of the alterations of the blink reflex in BSP, unless we assume that the somatosensory inputs use common interneurons to the trigemino-facial pathway for the elicitation of the blink reflex. An auditory stimulus elicits also a blink reflex and this has been found exaggerated in BSP patients (65). These authors recorded auditory startle responses (ASRs) from masseter, orbicularis oculi, sternocleidomastoid, and biceps brachii muscles and found abnormalities of different types (shortening latency, increased response probability, or enhanced response size in various of these muscles), pointing out to a general increase of response excitability to the auditory stimuli.

Patients with BSP have shown increased levels of activity in the orbicularis oculi in comparison to healthy subjects (66). One of the reasons for such increase in activity may be the visual stimuli acting on reflex contraction. Light is undoubtedly a source of discomfort in patients with BSP and, consequently, reduced eyelid contraction force has been reported together with increased comfort and reduction of overall light sensitivity and BSP frequency in patients with BSP using FL-41 tinted lenses (67).

## Hypotheses for Future Work

Many lines of research are of interest for further understanding the pathophysiological mechanisms of BSP. Various of them have been suggested along the previous paragraphs. In neuroimaging, it is apparent the shift of interest, driven out of building hypothesis on experience, from early sites of secondary dystonias in the basal ganglia through the involvement of the parietal cortex and the thalamus to the most recent hint on the cerebellar dysfunction. Certainly, all these structures may form a network that is dysfunctional in BSP, but the exact contribution of each and the overall cause of the dysfunction are still unknown. Tools to study cerebellar function in relation to the eyelid movements or orbicularis oculi contraction are very scarce. Recently, though, Ryan and coworkers (68) have suggested that eyelid conditioning and long-term depression of the blink reflex induced by a high frequency electrical discharge on the trigeminal nerve before elicitation of the blink reflex may share the same circuits and suggested that such high frequency stimulation may be a way to long-term depress trigeminal blink circuit activity in diseases like BSP. In fact, studies of eyeblink conditioning in BSP have not been reported so far, probably because of the difficulties related to interference by involuntary spasms.

An area deserving more study in BSP is the relationship between the antagonistic muscles in control of eyelid position. The orbicularis oculi muscle is easily accessible with surface EMG but the levator palpebrae muscle requires needle recording and the recording system itself is nowadays undoubtedly interfering with normal behavior. Eyelid position is controlled involuntarily by many sources, including the amount of light, tiredness, emotion, pain, and many other inputs. Likely, the levator palpebrae is tonically active most of the time, requiring some phasic contribution from the orbicularis oculi to refresh or reset the activity. A premovement silence of the tonic activity of the levator palpebrae may be needed for this muscle to have a burst of activity large enough to elevate the eyelid. The orbicularis oculi spasms may just be the result of unsuccessful attempts to reset the levator palpebrae, much as it has been described for the soleus when a standing person prepares for fast tip-toeing in a reaction time ask paradigm (69). How these mechanisms apply to BP is not yet fully understood but the fairly intimate relationship between the two muscles is certainly impaired in these patients.

Finally, another area in which research must be much rewarding for the care of BSP patients is the understanding of the role of eye diseases in the generation of BSP. A significant positive association between BSP and prior eye diseases has been reported by Defazio et al. (70). Symptoms of dry eye and other alterations are common in these patients but we do not know if they are consequent to, causative of, or concomitant with, BSP. Noxious stimuli may trigger reactions that are out of our conscious control. Awareness of them is not always easy and sensitization of circuits may take place even before the patient realizes that something is wrong. If we accept that this is one mechanism for the generation of BSP, we need early detection of possible eye disorders causing abnormal sensation or involuntary reactions to look for means to avoid consolidation of potentially abnormal circuits in case the disorder does not have a solution.

## Conclusion

There is much work to do to understand and treat BSP, ranging from muscle dysfunction to cerebral abnormalities. We do not know if some signs observed are cause or consequence of the disorder. The involuntary reaction of the body to a relatively small insult may modify circuits that later would lead to the complexity of symptoms characteristic of the disorder. We have to recognize the noxious stimulus that first triggered the process and separate it from the complex reactions leading to adaptation and compensation that finally lead to dystonia. The study of phenomenology can quantify and document the clinical expression of the disorder but this is not sufficient. Serious cooperation among various specialties is a must for the ophthalmologists to treat eye disorders, neurologists to detect early signs of abnormal behavior, psychologists to take care of emotional and cognitive disorders, rehabilitation experts to help generate beneficial plastic changes, and others.

## Author Contributions

JV-S and GD have contributed equally to the completion of this manuscript. Both have met and discussed on how to prepare the review. GD was more in charge of clinical and epidemiological aspects, while JV-S was more in charge of neurophysiology and neuroimaging.

## Conflict of Interest Statement

The authors declare that the research was conducted in the absence of any commercial or financial relationships that could be construed as a potential conflict of interest.

## References

[1] Marsden CD The problem of adult-onset idiopathic torsion dystonia and other isolated dyskinesias in adult life (including blepharospasm, oromandibular dystonia, dystonic writer’s cramp, and torticollis, or axial dystonia). Adv Neurol (1976) 14:259–76. 941774

[2] Marsden CD. Blepharospasm-oromandibular dystonia syndrome (Brueghel’s syndrome). A variant of adult-onset torsion dystonia? J Neurol Neurosurg Psychiatry (1976) 39:1204–9. 10.1136/jnnp.39.12.1204 1011031 PMC492566

[3] Defazio G Abbruzzese G Livrea P Berardelli A. Epidemiology of primary dystonia. Lancet Neurol (2004) 3:673–8. 10.1016/S1474-4422(04)00907-X 15488460

[4] Abbruzzese G Berardelli A Girlanda P Marchese R Martino D Morgante F Long-term assessment of the risk of spread in primary late-onset focal dystonia. J Neurol Neurosurg Psychiatry (2008) 79:392–6. 10.1136/jnnp.2007.124594 17635969

[5] Defazio G Conte A Gigante AF Fabbrini G Berardelli A. Is tremor in dystonia a phenotypic feature of dystonia? Neurology (2015) 84:1053–9. 10.1212/WNL.0000000000001341 25663232

[6] Martino D Liuzzi D Macerollo A Aniello MS Livrea P Defazio G. The phenomenology of the geste antagoniste in primary blepharospasm and cervical dystonia. Mov Disord (2010) 25:407–12. 10.1002/mds.23011 20108367

[7] Fabbrini G Defazio G Colosimo C Thompson PD Berardelli A. Cranial movement disorders: clinical features, pathophysiology, differential diagnosis and treatment. Nat Clin Pract Neurol (2009) 5:93–105. 10.1038/ncpneuro1006 19194389

[8] Bentivoglio AR Daniele A Albanese A Tonali PA Fasano A. Analysis of blink rate in patients with blepharospasm. Mov Disord (2006) 21:1225–9. 10.1002/mds.20889 16622858

[9] Conte A Defazio G Ferrazzano G Hallett M Macerollo A Fabbrini G Is increased blinking a form of blepharospasm? Neurology (2013) 80:2236–41. 10.1212/WNL.0b013e318296e99d 23751916 PMC3721097

[10] Defazio G Hallett M Jinnah HA Berardelli A. Development and validation of a clinical guideline for diagnosing blepharospasm. Neurology (2013) 81:236–40. 10.1212/WNL.0b013e31829bfdf6 23771487 PMC3770163

[11] Defazio G Hallett M Jinnah HA Stebbins GT Gigante AF Ferrazzano G Development and validation of a clinical scale for rating the severity of blepharospasm. Mov Disord (2015) 30:525–30. 10.1002/mds.26156 25847472 PMC4878674

[12] Martino D Defazio G Alessio G Abbruzzese G Girlanda P Tinazzi M Relationship between eye symptoms and blepharospasm: a multicenter case-control study. Mov Disord (2005) 20:1564–70. 10.1002/mds.20635 16092106

[13] Fabbrini G Berardelli I Moretti G Pasquini M Bloise M Colosimo C Psychiatric disorders in adult-onset focal dystonia: a case-control study. Mov Disord (2010) 25:459–65. 10.1002/mds.22983 20108377

[14] Conte A Berardelli I Ferrazzano G Pasquini M Berardelli A Fabbrini G. Non-motor symptoms in patients with adult-onset focal dystonia: sensory and psychiatric disturbances. Parkinsonism Relat Disord (2016) 22(Suppl 1):S111–4. 10.1016/j.parkreldis.2015.09.001 26360238

[15] Avanzino L Martino D Marchese R Aniello MS Minafra B Superbo M Quality of sleep in primary focal dystonia: a case-control study. Eur J Neurol (2010) 17:576–81. 10.1111/j.1468-1331.2009.02884.x 20039936

[16] Romano R Bertolino A Gigante A Martino D Livrea P Defazio G. Impaired cognitive functions in adult-onset primary cranial cervical dystonia. Parkinsonism Relat Disord (2014) 20:162–5. 10.1016/j.parkreldis.2013.10.008 24161376

[17] Khooshnoodi MA Factor SA Jinnah HA. Secondary blepharospasm associated with structural lesions of the brain. J Neurol Sci (2013) 331:98–101. 10.1016/j.jns.2013.05.022 23747003 PMC3732185

[18] Black KJ Ongür D Perlmutter JS. Putamen volume in idiopathic focal dystonia. Neurology (1998) 51:819–24. 10.1212/WNL.51.3.819 9748033

[19] Etgen T Mühlau M Gaser C Sander D. Bilateral grey-matter increase in the putamen in primary blepharospasm. J Neurol Neurosurg Psychiatry (2006) 77:1017–20. 10.1136/jnnp.2005.087148 16690695 PMC2077759

[20] Obermann M Yaldizli O De Greiff A Lachenmayer ML Buhl AR Tumczak F Morphometric changes of sensorimotor structures in focal dystonia. Mov Disord (2007) 22:1117–23. 10.1002/mds.21495 17443700

[21] Martino D Di Giorgio A D’Ambrosio E Popolizio T Macerollo A Livrea P Cortical gray matter changes in primary blepharospasm: a voxel-based morphometry study. Mov Disord (2011) 26:1907–12. 10.1002/mds.23724 21717508

[22] Horovitz SG Ford A Najee-Ullah MA Ostuni JL Hallett M. Anatomical correlates of blepharospasm. Transl Neurodegener (2012) 1:12. 10.1186/2047-9158-1-12 23210426 PMC3514098

[23] Ramdhani RA Kumar V Velickovic M Frucht SJ Tagliati M Simonyan K. What’s special about task in dystonia? A voxel-based morphometry and diffusion weighted imaging study. Mov Disord (2014) 29:1141–50. 10.1002/mds.25934 24925463 PMC4139455

[24] Lehéricy S Tijssen MA Vidailhet M Kaji R Meunier S. The anatomical basis of dystonia: current view using neuroimaging. Mov Disord (2013) 28:944–57. 10.1002/mds.25527 23893451

[25] Yang J Luo CY Song W Chen Q Chen K Chen XP Altered regional spontaneous neuronal activity in blepharospasm: a resting state fMRI study. J Neurol (2013) 260:2754–60. 10.1007/s00415-013-7042-8 23900755

[26] Smith D Ishikawa T Dhawan V Winterkorn JS Eidelberg D. Lid opening apraxia is associated with medial frontal hypometabolism. Mov Disord (1995) 10:341–4. 10.1002/mds.870100319 7651454

[27] Esmaeli-Gutstein B Nahmias C Thompson M Kazdan M Harvey J. Positron emission tomography in patients with benign essential blepharospasm. Ophthal Plast Reconstr Surg (1999) 15:23–7. 10.1097/00002341-199901000-00006 9949425

[28] Asanuma K Carbon-Correll M Eidelberg D. Neuroimaging in human dystonia. J Med Invest (2005) 52(Suppl):272–9. 10.2152/jmi.52.272 16366514

[29] Kerrison JB Lancaster JL Zamarripa FE Richardson LA Morrison JC Holck DE Positron emission tomography scanning in essential blepharospasm. Am J Ophthalmol (2003) 136:846–52. 10.1016/S0002-9394(03)00895-X 14597035

[30] Hutchinson M Nakamura T Moeller JR Antonini A Belakhlef A Dhawan V The metabolic topography of essential blepharospasm: a focal dystonia with general implications. Neurology (2000) 55:673–7. 10.1212/WNL.55.5.673 10980732

[31] Suzuki Y Mizoguchi S Kiyosawa M Mochizuki M Ishiwata K Wakakura M Glucose hypermetabolism in the thalamus of patients with essential blepharospasm. J Neurol (2007) 254:890–6. 10.1007/s00415-006-0468-5 17325818

[32] Murai H Suzuki Y Kiyosawa M Wakakura M Mochizuki M Ishiwata K Positive correlation between severity of blepharospasm and thalamic glucose metabolism. Case Rep Ophthalmol (2011) 2:50–4. 10.1159/000324459 22110436 PMC3219445

[33] Emoto H Suzuki Y Wakakura M Horie C Kiyosawa M Mochizuki M Photophobia in essential blepharospasm – a positron emission tomographic study. Mov Disord (2010) 25:433–9. 10.1002/mds.22916 20014062

[34] Schmidt KE Linden DE Goebel R Zanella FE Lanfermann H Zubcov AA. Striatal activation during blepharospasm revealed by fMRI. Neurology (2003) 60:1738–43. 10.1212/01.WNL.0000063306.67984.8C 12796523

[35] Baker RS Andersen AH Morecraft RJ Smith CD. A functional magnetic resonance imaging study in patients with benign essential blepharospasm. J Neuroophthalmol (2003) 23:11–5. 10.1097/00041327-200303000-00003 12616082

[36] Zhou B Wang J Huang Y Yang Y Gong Q Zhou D A resting state functional magnetic resonance imaging study of patients with benign essential blepharospasm. J Neuroophthalmol (2013) 33:235–40. 10.1097/WNO.0b013e31828f69e5 23636105

[37] Perlmutter JS Stambuk MK Markham J Black KJ McGee-Minnich L Jankovic J Decreased [18F]spiperone binding in putamen in idiopathic focal dystonia. J Neurosci (1997) 17:843–50. 8987805 10.1523/JNEUROSCI.17-02-00843.1997 PMC6573223

[38] Horie C Suzuki Y Kiyosawa M Mochizuki M Wakakura M Oda K Decreased dopamine D receptor binding in essential blepharospasm. Acta Neurol Scand (2009) 119:49–54. 10.1111/j.1600-0404.2008.01053.x 18540899

[39] Basso MA Powers AS Evinger C An explanation for reflex blink hyperexcitability in Parkinson’s disease. I. Superior colliculus. J Neurosci (1996) 16:7308–17. 8929437 10.1523/JNEUROSCI.16-22-07308.1996 PMC6578952

[40] Basso MA Evinger C An explanation for reflex blink hyperexcitability in Parkinson’s disease. II Nucleus raphe magnus. J Neurosci (1996) 16:7318–30. 8929438 10.1523/JNEUROSCI.16-22-07318.1996 PMC6578942

[41] Valls-Solé J Assessment of excitability in brainstem circuits mediating the blink reflex and the startle reaction. Clin Neurophysiol (2012) 123:13–20. 10.1016/j.clinph.2011.04.029 22030138

[42] Aramideh M Bour LJ Koelman JH Speelman JD Ongerboer de Visser BW. Abnormal eye movements in blepharospasm and involuntary levator palpebrae inhibition. Clinical and pathophysiological considerations. Brain (1994) 117:1457–74. 10.1093/brain/117.6.1457 7820580

[43] Esteban A Traba A Prieto J. Eyelid movements in health and disease. The supranuclear impairment of the palpebral motility. Neurophysiol Clin (2004) 34:3–15. 10.1016/j.neucli.2004.01.002 15030796

[44] Valls-Solé J. Electrodiagnostic studies of the facial nerve in peripheral facial palsy and hemifacial spasm. Muscle Nerve (2007) 36:14–20. 10.1002/mus.20770 17410591

[45] Valls-Solé J Chapter 19: the blink reflex and other cranial nerve reflexes. In: Aminoff’s MJ, editor. Electrodiagnosis in Clinical Neurology. San Francisco: Elsevier (2012). p. 421–35.

[46] Yeomans JS Li L Scott BW Frankland PW. Tactile, acoustic and vestibular systems sum to elicit the startle reflex. Neurosci Biobehav Rev (2002) 26:1–11. 10.1016/S0149-7634(01)00057-4 11835980

[47] Berardelli A Cruccu G Kimura J Ongerboer de Visser BW Valls-Solé J The orbicularis oculi reflexes. The International Federation of Clinical Neurophysiology. Electroencephalogr Clin Neurophysiol Suppl (1999) 52:249–53. 10590992

[48] Kimura J Disorders of interneurons in parkinsonism. The orbicularis oculi reflex to paired stimuli. Brain (1973) 96:87–96. 10.1093/brain/96.1.87 4695726

[49] Schwingenschuh P Katschnig P Edwards MJ Teo JT Korlipara LV Rothwell JC The blink reflex recovery cycle differs between essential and presumed psychogenic blepharospasm. Neurology (2011) 76:610–4. 10.1212/WNL.0b013e31820c3074 21321334 PMC3053342

[50] Graham FK The more or less startling effects of weak prestimulation. Psychophysiology (1975) 12:238–48. 10.1111/j.1469-8986.1975.tb01284.x 1153628

[51] Inglis WL Winn P The pedunculo-pontine tegmental nucleus: where the striatum meets the reticular formation. Prog Neurobiol (1995) 47:1–29. 10.1016/0301-0082(95)00013-L 8570851

[52] Gómez-Wong E Martí MJ Tolosa E Valls-Solé J. Sensory modulation of the blink reflex in patients with blepharospasm. Arch Neurol (1998) 55:1233–7. 10.1001/archneur.55.9.1233 9740118

[53] Valls-Solé J Muñoz JE Valldeoriola F. Abnormalities of prepulse inhibition do not depend on blink reflex excitability: a study in Parkinson’s disease and Huntington’s disease. Clin Neurophysiol (2004) 115:1527–36. 10.1016/j.clinph.2004.02.014 15203054

[54] Schicatano EJ Basso MA Evinger C. Animal model explains the origins of the cranial dystonia benign essential blepharospasm. J Neurophysiol (1997) 77:2842–6. 9163399 10.1152/jn.1997.77.5.2842

[55] Evinger C. Animal models for investigating benign essential blepharospasm. Curr Neuropharmacol (2013) 11:53–8. 10.2174/157015913804999441 23814538 PMC3580792

[56] Chuke JC Baker RS Porter JD. Bell’s Palsy-associated blepharospasm relieved by aiding eyelid closure. Ann Neurol (1996) 39:263–8. 10.1002/ana.410390217 8967759

[57] Manca D Muñoz E Pastor P Valldeoriola F Valls-Solé J. Enhanced gain of blink reflex responses to ipsilateral supraorbital nerve afferent inputs in patients with facial nerve palsy. Clin Neurophysiol (2001) 112:153–6. 10.1016/S1388-2457(00)00516-2 11137673

[58] Blomstedt P Tisch S Hariz MI. Pallidal deep brain stimulation in the treatment of Meige syndrome. Acta Neurol Scand (2008) 118:198–202. 10.1111/j.1600-0404.2008.00999.x 18336624

[59] Reese R Gruber D Schoenecker T Bäzner H Blahak C Capelle HH Long-term clinical outcome in Meige syndrome treated with internal pallidum deep brain stimulation. Mov Disord (2011) 26:691–8. 10.1002/mds.23549 21312284

[60] Limotai N Go C Oyama G Hwynn N Zesiewicz T Foote K Mixed results for GPi-DBS in the treatment of cranio-facial and cranio-cervical dystonia symptoms. J Neurol (2011) 258:2069–74. 10.1007/s00415-011-6075-0 21553081

[61] Foote KD Sanchez JC Okun MS. Staged deep brain stimulation for refractory craniofacial dystonia with blepharospasm: case report and physiology. Neurosurgery (2005) 56:E415. 10.1227/01.NEU.0000147978.67424.42 15670394

[62] Vagefi MR Lin CC McCann JD Anderson RL. Exacerbation of blepharospasm associated with craniocervical dystonia after placement of bilateral globus pallidus internus deep brain stimulator. Mov Disord (2008) 23:454–6. 10.1002/mds.21889 18074391

[63] Miwa H Nohara C Hotta M Shimo Y Amemiya K. Somatosensory-evoked blink response: investigation of the physiological mechanisms. Brain (1998) 121:281–91. 10.1093/brain/121.2.281 9549506

[64] Benbir G Kiziltan ME Blink reflex studies in blepharospasm: trigeminal and extratrigeminal somatosensory stimulation. J Clin Neurophysiol (2014) 31:535–40. 10.1097/WNP.0000000000000095 25462139

[65] Müller J Rinnerthaler M Poewe W Kofler M. Auditory startle reaction in primary blepharospasm. Mov Disord (2007) 22:268–72. 10.1002/mds.21270 17149731

[66] Berardelli A Rothwell JC Day BL Marsden CD. Pathophysiology of blepharospasm and oromandibular dystonia. Brain (1985) 108:593–608. 10.1093/brain/108.3.593 4041776

[67] Blackburn MK Lamb RD Digre KB Smith AG Warner JE McClane RW FL-41 tint improves blink frequency, light sensitivity, and functional limitations in patients with benign essential blepharospasm. Ophthalmology (2009) 116:997–1001. 10.1016/j.ophtha.2008.12.031 19410958 PMC2701948

[68] Ryan M Kaminer J Enmore P Evinger C. Trigeminal high-frequency stimulation produces short- and long-term modification of reflex blink gain. J Neurophysiol (2014) 111:888–95. 10.1152/jn.00667.2013 24285868 PMC3921395

[69] Nardone A Schieppati M. Postural adjustments associated with voluntary contraction of leg muscles in standing man. Exp Brain Res (1988) 69:469–80. 10.1007/BF00247301 3371431

[70] Defazio G Abbruzzese G Aniello MS Bloise M Crisci C Eleopra R Environmental risk factors and clinical phenotype in familial and sporadic primary blepharospasm. Neurology (2011) 77:631–7. 10.1212/WNL.0b013e3182299e13 21775731

